# Synthesis, Characterization, and Thermal Study of Divalent Germanium, Tin, and Lead Triazenides as Potential Vapor Deposition Precursors

**DOI:** 10.1021/acs.inorgchem.1c00695

**Published:** 2021-08-06

**Authors:** Rouzbeh Samii, David Zanders, Anton Fransson, Goran Bačić, Sean T. Barry, Lars Ojamäe, Vadim Kessler, Henrik Pedersen, Nathan J. O’Brien

**Affiliations:** † Department of Physics, Chemistry and Biology, Linköping University, Linköping SE-581 83, Sweden; ‡ Department of Chemistry, Carleton University, 1125 Colonel By Drive, Ottawa, Ontario K1S5B6, Canada; § Faculty of Chemistry and Biochemistry, Ruhr University Bochum, Universitätsstraße 150, Bochum 44801, Germany; ∥ Department of Molecular Sciences, Swedish University of Agricultural Sciences, P.O. Box 7015, Uppsala 75007, Sweden

## Abstract

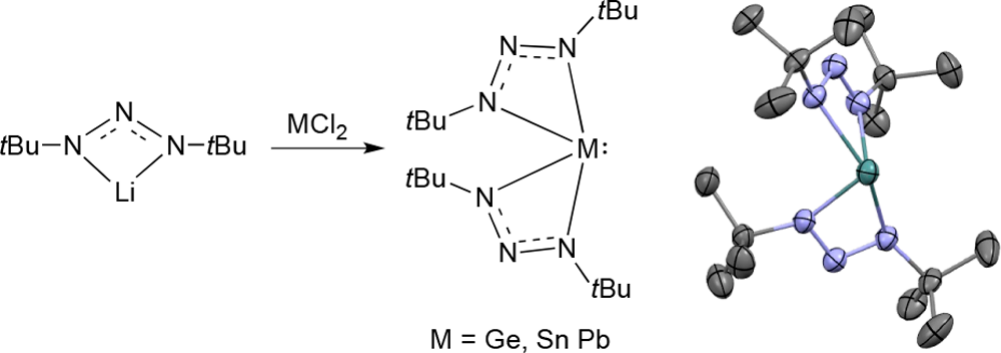

Only a few M–N bonded divalent group 14 precursors are available for vapor deposition, in particular for Ge and Pb. A majority of the reported precursors are dicoordinated with the Sn(II) amidinates, the only tetracoordinated examples. No Ge(II) and Pb(II) amidinates suitable for vapor deposition have been demonstrated. Herein, we present tetracoordinated Ge(II), Sn(II), and Pb(II) complexes bearing two sets of chelating 1,3-di-*tert*-butyltriazenide ligands. These compounds are thermally stable, sublime quantitatively between 60 and 75 °C (at 0.5 mbar), and show ideal single-step volatilization by thermogravimetric analysis.

## Abstract 2

Only a few tetracoordinated M−N bonded vapor deposition precursors exist for divalent group 14 elements. Although there are a couple of volatile tetracoordinated tin(II) amidinates, no such Ge and Pb compounds are suitable. Herein, we present volatile group 14 triazenides as potential vapor deposition precursors. These compounds are thermally stable, sublime quantitatively between 65 and 70 °C (0.5 mbar), and show single-step volatilization curves by thermogravimetric analysis.

## Introduction

**Figure 1:**
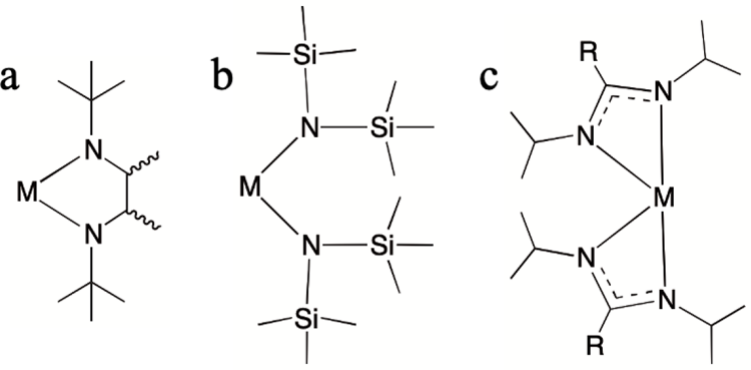
General structures of divalent group 14 (a) cyclic diamide, (b) hexamethyldisilazide, and (c) amidinates, where M = Ge, Sn, and Pb. For formamidinate and amidinates, R = H and CH_3_, respectively. These are the M–N bonded compound classes that have been utilized as ALD precursors for depositing group 14 chalcogenides.

Group 14 chalcogenides are of interest because of their outstanding electrical and optical properties.^1^ In particular, monochalcogenides such as germanium sulfide (GeS),^2^ germanium selenide (GeSe),^2^ tin sulfide (SnS),^2−9^ tin selenide (SnSe)^2,10−12^ and lead sulfide (PbS)^13−19^ have potential applications in future optoelectronics and photovoltaics. Manufacturing these electronic devices requires highly controlled deposition of conformal, dense, and defect-free films with low impurity levels. High-quality thin films are commonly grown by chemical vapor deposition (CVD) methods. One subset of CVD is atomic layer deposition (ALD), which is a low-temperature alternative that introduces the metal and nonmetal precursors into the reaction chamber separately in alternating pulses.^20^ This separation allows the deposition process to be governed exclusively by self-limiting chemical reactions on the growth surface. To obtain high-quality films by ALD, it is important to have metal and nonmetal precursors that possess favorable physical and chemical properties.^21^ An ALD precursor must be sufficiently volatile for gas-phase transport to the growth region. It should not decompose in the gas phase in a manner that leads to continuous CVD growth.^22^ At the surface, the precursor should react in a self-limiting manner to form a stable monolayer and easy-to-purge byproducts.^23^ After purging, the monolayer should react with a second precursor (e.g. H_2_S and H_2_O) in a second self-limiting reaction. To maximize film growth, the precursor should employ small ligands for effective surface saturation and dense coverage.^24^ Furthermore, using precursors of the same oxidation state as the target material removes the need for redox chemistry during deposition. Not having to rely on redox chemistry for the deposition process reduces the risk of forming undesired mixed-phase materials. Precursors with M–N bonds are desirable for ALD because of the high reactivity of the polarized bond. To date, there is only a small library of exclusively M–N bonded Ge(II), Sn(II), and Pb(II) precursors suitable for ALD. Figure 1 shows the general structures of some precursors used for growing group 14 monochalcogenides that are discussed.

The dicoordinated Ge(II) cyclic diamide and hexamethyldisilazide compounds have been used to deposit GeS^25^ and GeSe^26^ by ALD, respectively. However, the deposited GeS was X-ray amorphous, while the GeSe films contained unwanted carbon impurities. SnS has been demonstrated by ALD using the dicoordinated cyclic diamide and, tetracoordinated Sn(II) formamidinate and amidinate precursors.^27,28^ The amidinate ligand system provides tetracoordinated, thermally stable, and volatile precursors that afford stochiometric SnS with low levels of impurities.

Recently, PbS was deposited using the dicoordinated Pb–N bonded hexamethyldisilazide and cyclic diamide precursors, rendering near stochiometric films with low levels of carbon impurities.^29^ However, the deposition temperature for ALD growth was limited to ≤155 °C due to the poor thermal stability of the deposited surface species. Volatility data have not been reported for the tetracoordinated Ge(II) and Pb(II) amidinates in the literature.^30,31^ There are no other exclusively M–N bonded tetracoordinated Ge(II) and Pb(II) compounds used as precursors for ALD, as far as we know.

A ligand system closely related to the amidinates is the triazenide, where the endocyclic C is replaced by N. There are reports of tetracoordinated divalent group 14 triazenides.^32^ There is no mention of their volatility, and these compounds are most likely not suitable for vapor deposition because of their bulky 1,3-bis(2,6-diisopropylphenyl)triazenide ligand. Recently, we explored the 1,3-dialkyltriazenide ligand for group 13 metals and used the In(III) and Ga(III) 1,3-diisopropyltriazenide precursors for ALD of InN and GaN.^33−35^ To further explore the 1,3-dialkyltriazenide ligand, we envisaged its ability to stabilize divalent group 14 elements. This would potentially give a new class of tetracoordinated M–N bonded group 14 precursors for ALD. Herein, we present the synthesis, characterization, and thermal study of Ge(II), Sn(II), and Pb(II) compounds bearing two sets of the 1,3-di-*tert*-butyltriazenide ligand. Their high volatility and thermal stability make these group 14 triazenides interesting for use as ALD precursors.

## Results and Discussion

### Synthesis and Characterization of Triazenides **1**–**3**

**Scheme 1:**
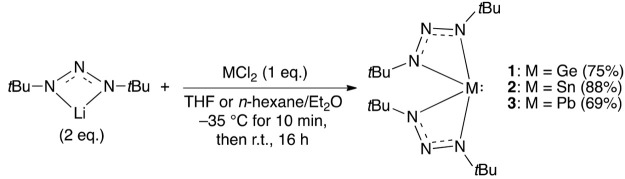
General Procedure for Making **1**–**3** The synthesis of **1** was carried out in tetrahydrofuran (THF), while the syntheses of **2** and **3** were carried out in a 1:4 mixture of diethyl ether (Et_2_O) and *n*-hexane.

The reaction of (1,3-di-*tert*-butyltriazenide)lithium with MCl_2_, where M = Ge, Sn, or Pb, gave **1**–**3** in good yields after recrystallization (Scheme 1; CCDC 2058914, 2058915, and 2058916 for **1**–**3**, respectively). Compounds **1**–**3** are air- and moisture-sensitive and low-melting yellow solids. The compounds were characterized by NMR spectroscopy, X-ray crystallography, electron impact mass spectrometry, elemental analysis, and melting point measurement.

The ^1^H NMR spectra of compounds **1**–**3** showed a singlet between 1.30 and 1.40 ppm, indicating fast exchange for all protons of the *tert*-butyl groups. The ^13^C NMR spectra of **1**–**3** gave the primary and quaternary C peaks at 30.5–30.6 and 58.9–61.1 ppm, respectively. All NMR spectra are presented in the Supporting Information.

**Figure 2:**
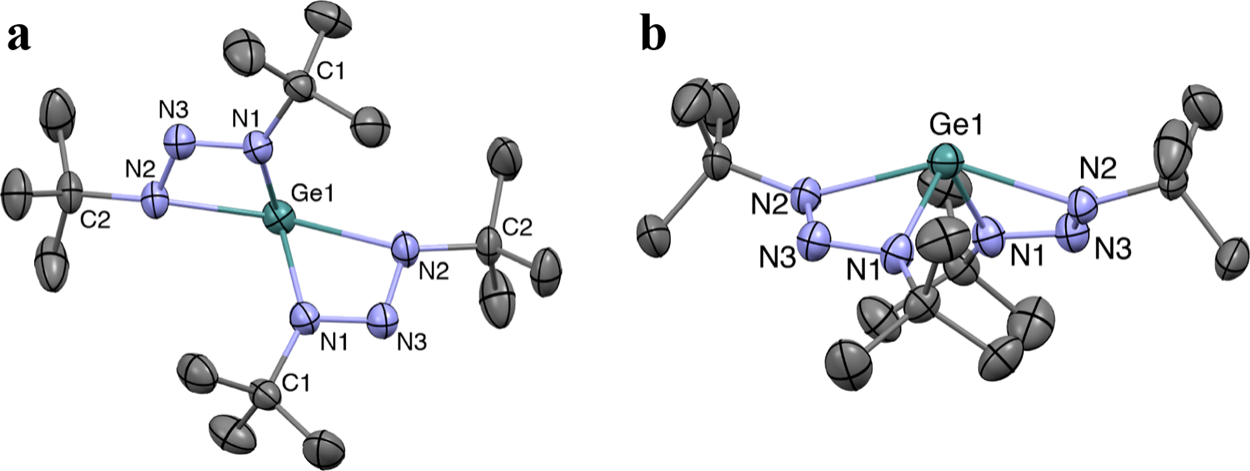
Crystal structure of **1** viewed (a) parallel and (b) perpendicular to the stereochemically active lone pair residing on the Ge atom. Thermal ellipsoids are depicted at the 50% probability level, and all H atoms were removed for clarity.

The crystal structure of **1** showed two chelating 1,3-di-*tert*-butyltriazenide ligands on the metal center (Figure 2). Both ligands are forced to the same side of the metal center by a stereochemically active lone pair, situated on the opposite side (Figure 2b). The coordination of the Ge atom is best described as a distorted seesaw geometry, as suggested by a geometry index (τ′_4_) of 0.65.^36^ See the Supporting Information for the geometry index calculations for compounds **1**–**3** and the crystal structures of compounds **2** and **3**. Compounds **2** and **3** have crystal structures and molecular geometries analogous to that of **1**. However, the Sn and Pb centers are further distorted toward tetrahedral geometry (τ′_4_ = 0.82 and 0.86 for **2** and **3**, respectively). The β angle decreases in the order **1** > **2** > **3**. This trend can be explained by a greater p character of the M–N bond when moving down the group (Table 2).

The geometries of **1**–**3** are in good agreement with previously reported analogs **1A**–**3A**,^32^ which employ the bulkier 2,6-diisopropylphenyl R groups in place of *tert*-butyl (Tables 1 and 2).

**Table 1:**
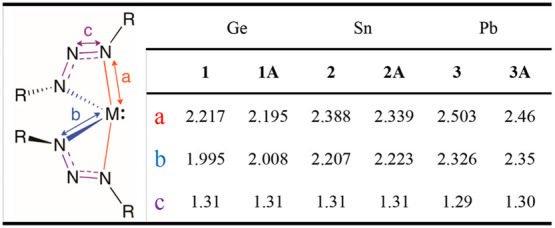
Average Bond Lengths (Å) for **1**–**3** (R = *tert*-butyl) and Their Respective Analogs **1A**–**3A** (R = 2,6-diisopropylphenyl)

**Table 2:**
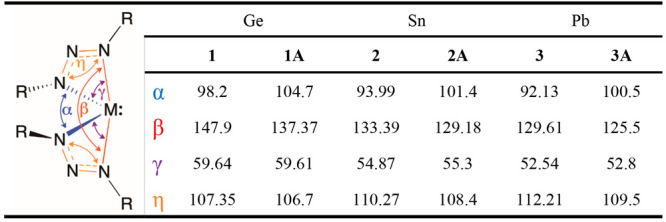
Average Bond Angles (deg) for **1**–**3** (R = *tert*-butyl) and Their Respective Analogs **1A**–**3A** (R = 2,6-diisopropylphenyl)

**Figure 3:**
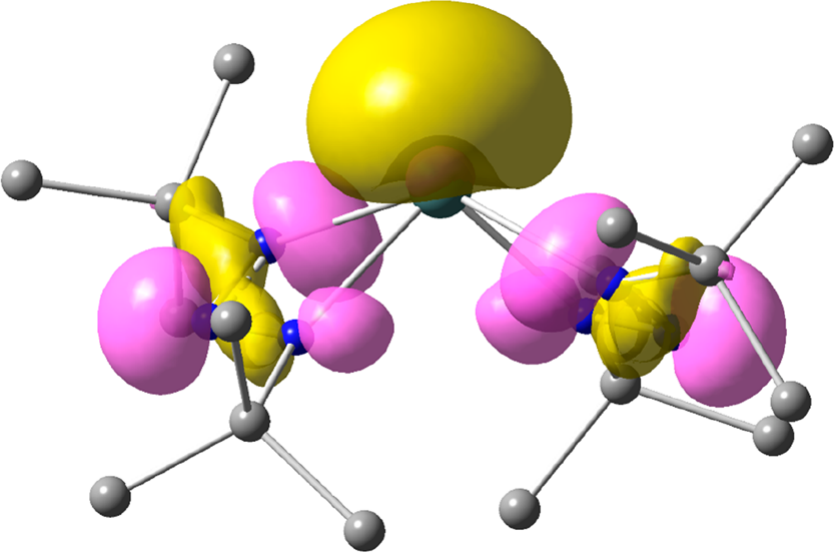
HOMO for **1** obtained from DFT calculations. H atoms are omitted for clarity. The stereochemically active lone pair occupies a large part of the coordination sphere of the metal center.

The geometries of **1**–**3** obtained from quantum-chemical density functional theory (DFT) computations are in good agreement with their crystal structures (see the Supporting Information). The stereochemically active lone pair resides in the highest occupied molecular orbital (HOMO; displayed for **1** in Figure 3).

### Thermal Properties of Triazenides **1**–**3**

**Figure 4:**
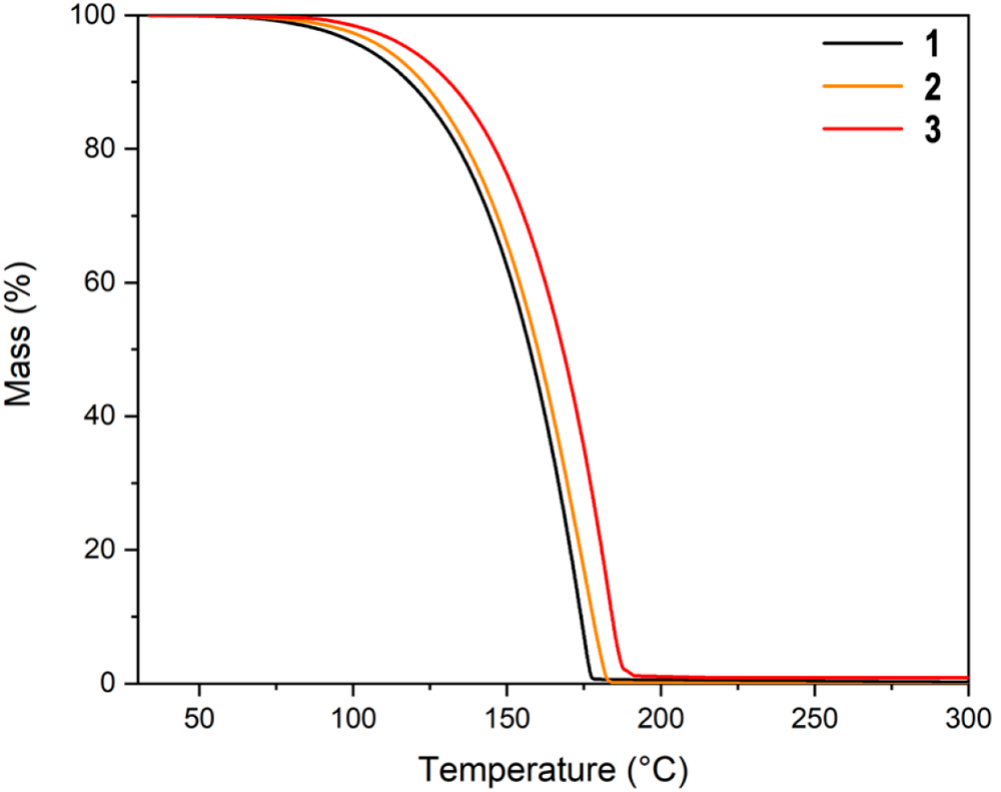
TGA of compounds **1**–**3** (10 mg samples) showing near-ideal single-step volatilization with negligible residual mass.

Thermogravimetric analysis (TGA) showed **1**–**3** volatilize in a single-step with onset temperatures between 137 and 152 °C, and negligible residual mass (Figure 4 and Table 3). Compounds **1** and **3** are therefore the first examples of volatile tetracoordinated M–N bonded Ge(II) and Pb(II) compounds, making them potentially useful as vapor deposition precursors. When comparing TGA data, compound **2** appears only slightly less volatile than Sn(II) formamidinate.^28^ This result is noteworthy as Sn(II) formamidinate is the most volatile tetracoordinated M–N bonded compound to date. The TGA data show **1**–**3** are highly volatile, exhibiting 1 Torr of vapor pressures between ∼90 and 100 °C (Table 3). Compounds **1**–**3** sublime quantitatively between 60 and 75 °C under reduced pressure (0.5 mbar).

**Table 3:** Summarized TGA and DSC Results for **1**–**3**

	DSC decomp exotherm onset (°C)	onset of volatilization (°C)	residual mass (%)	temp at 1 Torr of vapor pressure (°C)	sublimation temp (°C)^1^
**1**	181	137	0	87	60
**2**	280	140	0	96	65
**3**	170	152	0	103	75

1 Sublimations were performed at 0.5 mbar of pressure.

Differential scanning calorimetry (DSC) of **1** showed overlapping exotherms at 181 °C indicated by the irregular peak shape. The compound degraded when heated to 200 °C in a sealed capillary, suggesting the exotherm is a decomposition event. Compound **2** gave an exotherm at 175 °C, preceded by a small peak at 160 °C. However, it remained intact when heated to 200 °C for 24 h, indicating the exotherms at 160 °C and 175 °C are not decompositon events. A third exotherm was found at 280 °C and is most likely a decomposition event as the compound released gas bubbles during melting and changed from a yellow liquid to an off-white solid when heated to 275 °C in a sealed capillary. Compound **3** showed an exotherm at 85 °C, but the melt resolidified, and the melting point was unaffected by repeated heating of the compound to 110 °C for hours in sealed capillaries. The heat-treated compound remained unchanged from pure **3**, both visually and by ^1^H NMR analysis, suggesting the exotherm at 85 °C is not a decomposition event. A second exotherm of sharp overlapping signals was found at 170 °C. This exotherm at 170 °C is most likely a decomposition event as the compound darkened when heated above 180 °C in a sealed capillary. Upon cooling, the melt resolidified into an inhomogeneous, yellow and gray solid. A third exotherm was observed between 270 and 300 °C. The sublimation temperatures of **1**–**3** and their TGA and DSC data are summarized in Table 3. See the Supporting Information for individual TGA and DSC data.

Disregarding the first exotherm for **3**, compounds **1**–**3** have onsets of decomposition from DSC well above their respective onsets of volatilization from TGA. DSC revealed no thermal events occurring for **1** and **2** at temperatures <100 °C, consistent with the observations during melting point measurements.

## Conclusions

In summary, we have presented volatile divalent Ge, Sn, and Pb triazenides as potential precursors for vapor deposition. To our knowledge, **1** and **3** are the first examples of tetracoordinated and exclusively M–N bonded Ge(II) and Pb(II) compounds with high volatility and thermal stability. The compounds sublime quantitatively between 60 and 75 °C (at 0.5 mbar), and show near-ideal volatilization by TGA with onsets between ∼130 and 160 °C.

## Experimental Section

### General Experimental Procedures

***Caution!** Because catenated nitrogen compounds are known to be associated with explosive hazards, tert-butylazide and compounds **1**–**3** are possible explosive energetic materials. Although we have not experienced any problems in the synthesis, characterization, sublimation, heating, and handling of compounds **1**–**3**, their energetic properties have not been fully investigated and are therefore unknown. We therefore highly recommend all appropriate standard safety precautions for handling explosive materials (safety glasses, face shield, blast shield, leather gloves, polymer apron, and ear protection) always be used when working with tert-butylazide and compounds **1**–**3**. In addition, keep tert-butylazide and compounds **1**–**3** away from other metals (Cu and Ag) and Hg bubblers while using Schlenk equipment.*

All reactions and manipulations were carried out under a N_2_ atmosphere, either on a Schlenk line using Schlenk air-free techniques or in a dry box. All anhydrous solvents were purchased from Sigma-Aldrich and further dried over molecular sieves 4 Å. The GeCl_2_·dioxane complex, SnCl_2_ (98%) and PbCl_2_ (98%), and a *tert*-butyllithium solution (1.7 M in pentane) were purchased from Sigma-Aldrich and used without further purification. The preparation of *tert*-butylazide was undertaken according to the literature procedure.^37^ All NMR spectra were measured with Oxford Varian 300 and AS500 spectrometers at room temperature unless otherwise stated. Solvent peaks were used as internal standards for ^1^H NMR (300 and 500 MHz) and ^13^C NMR (75 and 125 MHz). Electron ionization mass spectrometry (EI-MS) was performed with a Kratos Concept–Magnetic sector mass spectrometer in high-resolution mode. Melting points were determined in sealed capillaries with a Stuart SMP10 melting point apparatus and are uncorrected. Elemental analyses were performed by Mikroanalytisches Laboratorium Kolbe, Germany.

### Synthesis of the Triazenide Ligand and Compounds **1**–**3**

#### Lithium 1,3-Di-*tert*-butyltriazenide Ligand

*tert*-Butyllithium (1.7 M in pentane, 110 mL, 187 mmol) was added dropwise to a solution of *tert*-butylazide (18.6 g, 188 mmol) in *n*-hexane (150 mL) at −78 °C. The reaction mixture was stirred for 30 min and then allowed to warm to room temperature for 1 h. The reaction mixture was then concentrated under reduced pressure to give a light-yellow solid (29.7 g, 97%). This solid was of sufficient purity to be used in the subsequent reactions without further purification. A small amount was purified for characterization by recrystallization from *n*-hexane at −35 °C to give a solid. Colorless solid, mp: 247−250 °C. ^1^H NMR (300 MHz, C_6_D_6_): δ 1.28 (s, CH_3_, 18H). ^13^C{^1^H} NMR (75 MHz, C_6_D_6_): δ 30.7 (s, CH_3_), 56.7 (s, C_q_). Anal. Calcd for C_8_H_18_LiN_3_: C, 58.88; H, 11.12; N, 25.75. Found: C, 58.88; H, 11.14; N, 25.67.

#### Bis(1,3-di-*tert*-butyltriazenide)germanium(II) (**1**)

A solution of (1,3-di-*tert*-butyltriazenide)lithium(I) (3.83 g, 23.5 mmol) in THF (20 mL) at −35 °C was added dropwise to a solution of GeCl_2_·dioxane (2.73 g, 11.7 mmol) in THF (30 mL) at −35 °C. The reaction mixture was held at −35 °C for 10 min and then stirred at room temperature for 16 h. The reaction mixture was concentrated under reduced pressure, and the resulting residue was suspended in *n*-hexane, filtered through a pad of Celite, and concentrated under reduced pressure to give the crude product. The crude product was purified by recrystallization from THF/acetonitrile (MeCN) at −35 °C to give **1** as a solid (3.39 g, 75%).

**1**: Pale-yellow solid, mp: 82−84 °C. Sublimation: ∼60 °C (0.5 mbar). ^1^H NMR (300MHz, C_6_D_6_): δ 1.40 (s, 36H, CH_3_). ^13^C{^1^H} NMR (125 MHz, C_6_D_6_): δ 30.6 (s, CH_3_), 59.1 (s, C_q_). EI-MS (LR): 230.1 (100%), 245.1 (<5%), 287.1 (<5%), 329.2 (25%), 386.2 (30%). Anal. Calcd for C_16_H_36_GeN_6_: C, 49.90; H, 9.42; N, 21.82. Found: C, 49.70; H, 9.40; N, 21.78.

#### Bis(1,3-di-*tert*-butyltriazenide)tin(II) (**2**)

A solution of (1,3-di-*tert*-butyltriazenide)lithium(I) (1.25 g, 7.65 mmol) in a 1:4 mixture of Et_2_O/*n*-hexane (5 mL) at −35 °C was added dropwise to a suspension of SnCl_2_ (0.17 g, 0.91 mmol) in *n*-hexane (5 mL) at −35 °C and then stirred at room temperature for 16 h. The reaction mixture was filtered through a pad of Celite and concentrated under reduced pressure to give the crude product. The crude product was purified by recrystallization from THF/MeCN at −35 °C to give **2** as a solid (0.79 g, 88%).

**2**: Yellow solid, mp: 58–59 °C. Sublimation: ∼65 °C (0.5 mbar). ^1^H NMR (500 MHz, C_6_D_6_): δ 1.35 (s, 36H, CH_3_). ^13^C{^1^H} NMR (125 MHz, C_6_D_6_): δ 30.6 (s, CH_3_), 58.9 (s, C_q_). EI-MS (LR): 134.9 (8%), 274.1 (8%), 373.1 (<5%), 432.2 (25%). Anal. Calcd for C_16_H_36_N_6_Sn: C, 44.57; H, 8.41; N, 19.49. Found: C, 44.04; H, 8.31; N, 19.23.

#### Bis(1,3-di-*tert*-butyltriazenide)lead(II) (**3**)

Compound **3** was synthesized in the same manner as **2** using a solution of (1,3-di-*tert*-butyltriazenide)lithium(I) (1.43 g, 8.76 mmol) in a 1:4 mixture of Et_2_O/*n*-hexane (20 mL) and a suspension of PbCl_2_ (1.22 g, 4.39 mmol) in *n*-hexane (20 mL). The crude product was purified by recrystallization from THF/MeCN at −35 °C to give **3** as a solid (1.58 g, 69%).

**3**: Yellow solid, mp: 83−84 °C. Sublimation: ∼75 °C (0.5 mbar). ^1^H NMR (500 MHz, C_6_D_6_): δ 1.30 (s, 36H, CH_3_). ^13^C{^1^H} NMR (125 MHz, C_6_D_6_): δ 30.5 (s, CH_3_), 61.1 (s, C). Anal. Calcd for C_16_H_36_N_6_Pb: C, 36.98; H, 6.98; N, 16.17. Found: C, 36.56; H, 6.97; N, 16.03.

### X-ray Crystallographic Analysis

Single crystals of **1**–**3** were obtained by recrystallization from THF/MeCN at −35 °C. The single crystals were used for X-ray diffraction data collection on a Bruker D8 SMART Apex-II diffractometer, using graphite-monochromated Mo Kα radiation (λ = 0.71073 Å) at 153 K. All data were collected in a hemisphere with over 95% completeness to 2θ < 50.05°. The structures were solved by direct methods. The coordinates of the metal atoms were determined from the initial solutions and the N and C atoms by subsequent differential Fourier syntheses. The solution did not contain much residual electron density, but it remained for a multitude of additional possible positions of light atoms. All non-H atoms were refined, first by isotropic and then by anisotropic approximation using Bruker *SHELXTL* software. The H atoms were added in a riding approximation and refined isotropically. The solutions of compounds **1** and **2** gave one B-level alert each, which was caused by one atom in each structure failing the Hirshfeld test. These two atoms had significantly bent thermal ellipsoids, potentially caused by more than one preferred orientation for these atoms. These ellipsoids showed no preferred orientation upon manual inspection. Selected crystal data are summarized below.

**1**: C_16_H_36_GeN_6_, *M* = 385.12, monoclinic, space group *P*2/*c*, *a* = 9.702(7) Å, *b* = 6.055(5) Å, *c* = 18.549(14) Å, α = 90°, β = 100.971(9)°, γ = 90°, *V* = 1069.6(14) Å^3^, *Z* = 2, *D*_c_ = 1.196 g cm^−3^, μ = 1.440 mm^−1^, *T* = 153 K, 6500 reflections measured, 1877 unique, final R1 [*I* > 2σ(*I*)] = 0.0254, wR2 (all data) = 0.0672, GOF = 1.040.

**2**: C_16_H_36_N_6_Sn, *M* = 431.20, orthorhombic, space group *Pbcn*, *a* = 12.732(5) Å, *b* = 13.049(5) Å, *c* = 13.693(6) Å, *V* = 2274.9(16) Å^3^, *Z* = 4, *D*_c_ = 1.259 g cm^−3^, μ = 1.131 mm^−1^, *T* = 153 K, 16479 reflections measured, 2051 unique, final R1 [*I* > 2σ(*I*)] = 0.0256, wR2 (all data) = 0.0696, GOF = 0.934.

**3**: C_16_H_36_N_6_Pb, *M* = 519.70, orthorhombic, space group *Pbcn*, *a* = 12.786(4) Å, *b* = 12.794(4) Å, *c* = 13.734(4) Å, *V* = 2246.7(12) Å^3^, *Z* = 4, *D*_c_ = 1.536 g cm^−3^, μ = 7.518 mm^−1^, *T* = 153 K, 15085 reflections measured, 1982 unique, final R1 [*I* > 2σ(*I*)] = 0.0236, wR2 (all data) = 0.0795, GOF = 0.936.

### TGA

TGA was performed on Pt pans with a TA Instruments Q50 analyzer housed in an MBraun Labmaster 130 dry box filled with N_2_ (99.998 % purity). Pt pans were cleaned by ultrasonication, first in dilute nitric acid (∼3 M), then water, and last 2-propanol. The pans were heated in air by a propane torch until red hot to remove any remaining impurities. During the TGA experiments of **3**, if the compound decomposes, then Pb(0) might form and, consequently, alloy with the Pt pans. To avoid potential alloying, pans used for TGA of **3** were coated with a 55 nm Al_2_O_3_ layer as a precaution. The coating was grown at 200 °C, using trimethylaluminum and water (0.1 s pulses and 8 s purges each) for 500 cycles.^38^ All TGA experiments were performed under a flow of ultrapure N_2_ (99.999%, 60 sccm). For ramp experiments, samples were heated to 500 °C at a rate of 10 °C min^−1^. The Langmuir vapor pressure equations for compounds **1**–**3** were derived from the TGA data with 10 mg of mass loading using a previously reported method^39^ and employing bis(2,2,6,6-tetramethyl-3,5-heptanedionato)copper(II) as the calibrant.^40^ Isothermal TGA were carried out at 90 and 110 °C. A heating rate of 40 °C min^−1^ was used to rapidly reach the desired temperature. The experiments were carried out with the same mass loadings as the ramp experiments. The onset of volatilization was defined as the intersection between the tangent lines of the plateau and slope.

### DSC Analysis

DSC experiments were performed using a TA Instruments Q10 instrument. Inside a glovebox, samples of 0.30 ± 0.03 mg of **1**–**3** were sealed in Al pans. Unless otherwise stated, all samples were heated to 400 °C at a rate of 10 °C min^−1^. N_2_ (99.998%) was used as the purge gas. Experiments were performed in triplicate with similar mass loadings to ensure validity of the recorded data.

### Quantum-Chemical Computations

All quantum-chemical DFT computations were performed using *Gaussian 16* software.^41^ Structural optimization and harmonic normal-mode vibrational calculations were done using the B3LYP method^42,43^ together with Grimme’s version 3 dispersion correction^44^ and the def2TZVP^45,46^ basis set. Minimized structures were confirmed to have no imaginary frequencies.

## Supporting Information Available

Characterization of the compounds and computational calculation details (PDF)

## Accession Codes

CCDC 2058914–2058916 contain the supplementary crystallographic data for this paper. These data can be obtained free of charge via www.ccdc.cam.ac.uk/data_request/cif, or by emailing data_request@ccdc.cam.ac.uk, or by contacting The Cambridge Crystallographic Data Centre, 12 Union Road, Cambridge CB2 1EZ, UK; fax: +44 1223 336033.
