# Supplementary material for: Synthesis, Characterization, and Thermal Study of Divalent Germanium, Tin, and Lead Triazenides as Potential Vapor Deposition Precursors
Source: Inorg Chem. 2021 Aug 6;60(17):12759–65. doi: 10.1021/acs.inorgchem.1c00695 (PMC8424630; doi:10.1021/acs.inorgchem.1c00695)
Supplement: Supplementary file 1 — ic1c00695_si_001.pdf [file ic1c00695_si_001.pdf]

**Supporting information**

**for**

**Synthesis, Characterization and Thermal Study of Divalent Germanium, Tin and Lead Triazenides as Potential Vapor Deposition Precursors**

Rouzbeh Samii,[a] David Zanders,[b],[c] Anton Fransson,[a] Goran Bačić,[b] Sean T. Barry,[b] Lars Ojamäe,[a] Vadim Kessler,[d] Henrik Pedersen,[a] and Nathan J. O'Brien*,[a]

[a] Department of Physics, Chemistry and Biology, Linköping University, SE-581 83 Linköping, Sweden

[b] Department of Chemistry, Carleton University, 1125 Colonel By Drive, Ottawa, Ontario, K1S5B6, Canada

[c] Faculty of Chemistry and Biochemistry, Ruhr University Bochum, Universitätsstraße 150, 44801, Bochum, Germany

[d] Department of Molecular Sciences, Swedish University of Agricultural Sciences, P.O. Box 7015, 75007 Uppsala, Sweden

*Corresponding author: nathan.o.brien@liu.se

Table of contents

| | |
|---|---|
| NMR Spectra | S1-4 |
| Thermogravimetric Analysis | S5-6 |
| Differential Scanning Calorimetry | S7 |
| EI-MS Data | S8 |
| X-ray Crystallographic Analysis | S9-11 |
| Computational Calculations | S12-13 |

## NMR Spectra

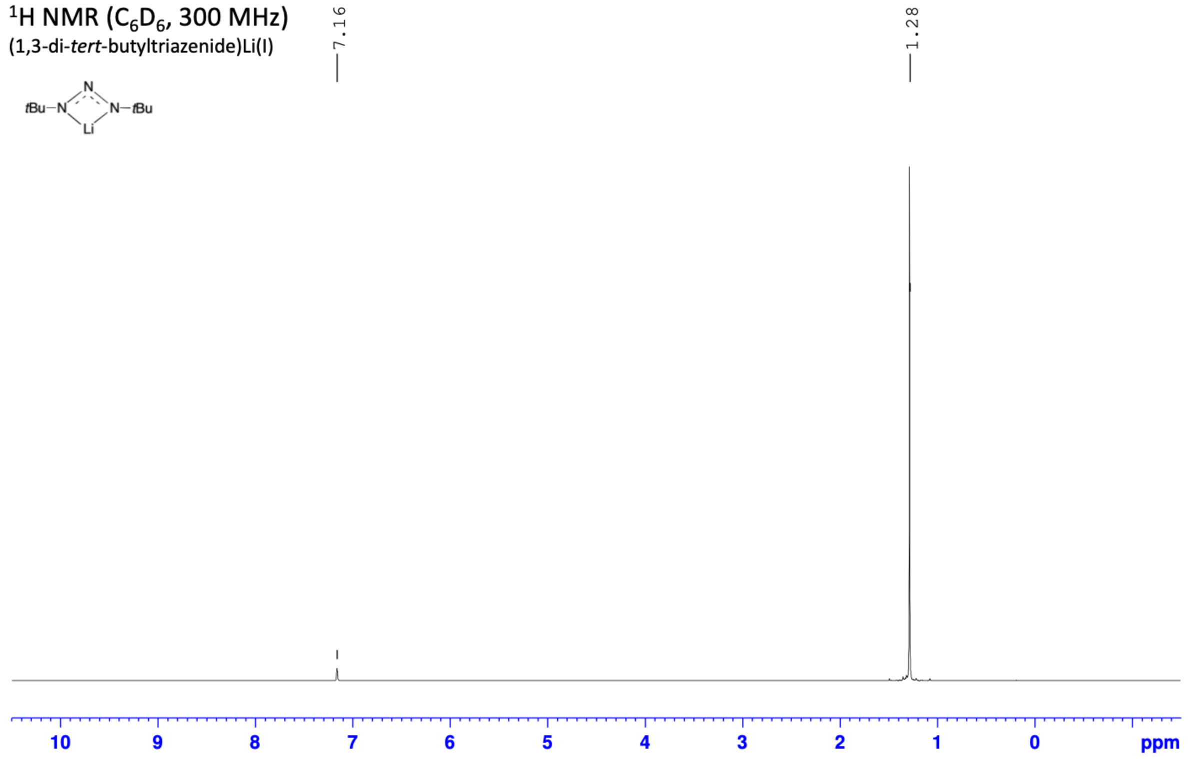


Figure S1: $^{1}$H NMR (300 MHz) spectrum of (1,3-di-*tert*-butyltriazenide)lithium(I) in $C_6D_6$.

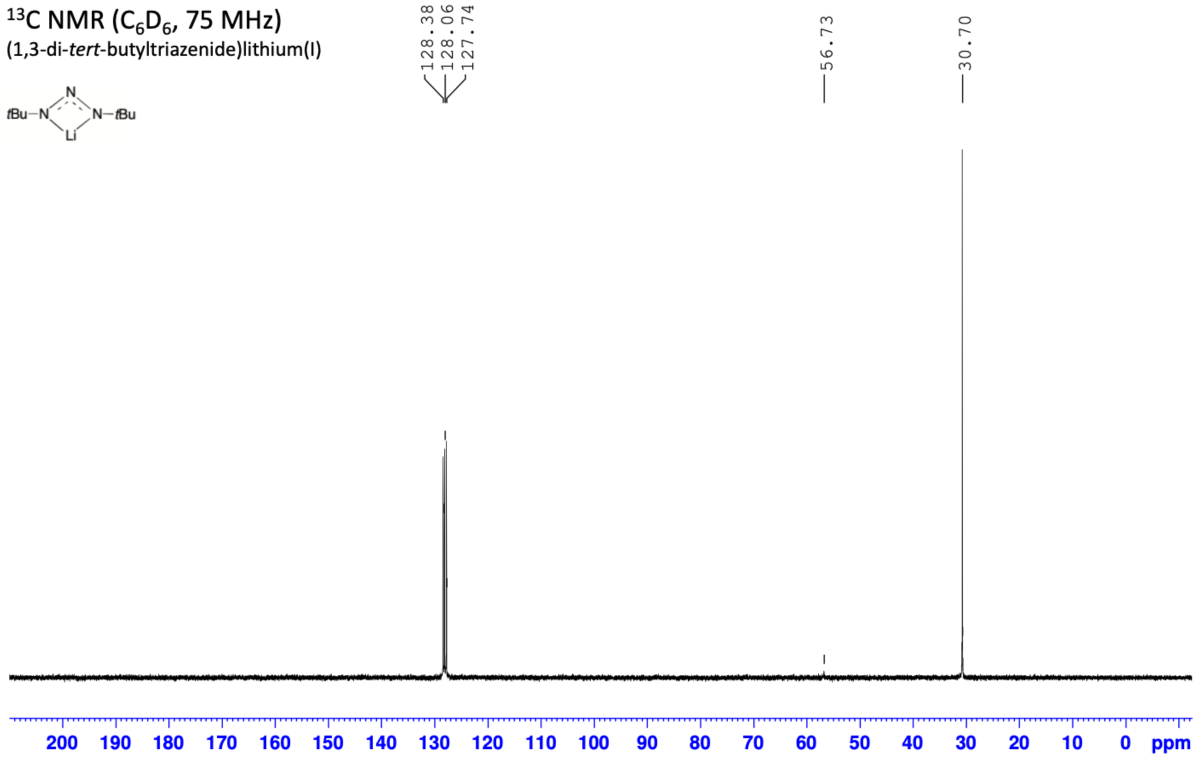


Figure S2: $^{13}$C NMR (75 MHz) spectrum of (1,3-di-*tert*-butyltriazenide)lithium(I) in $C_6D_6$.

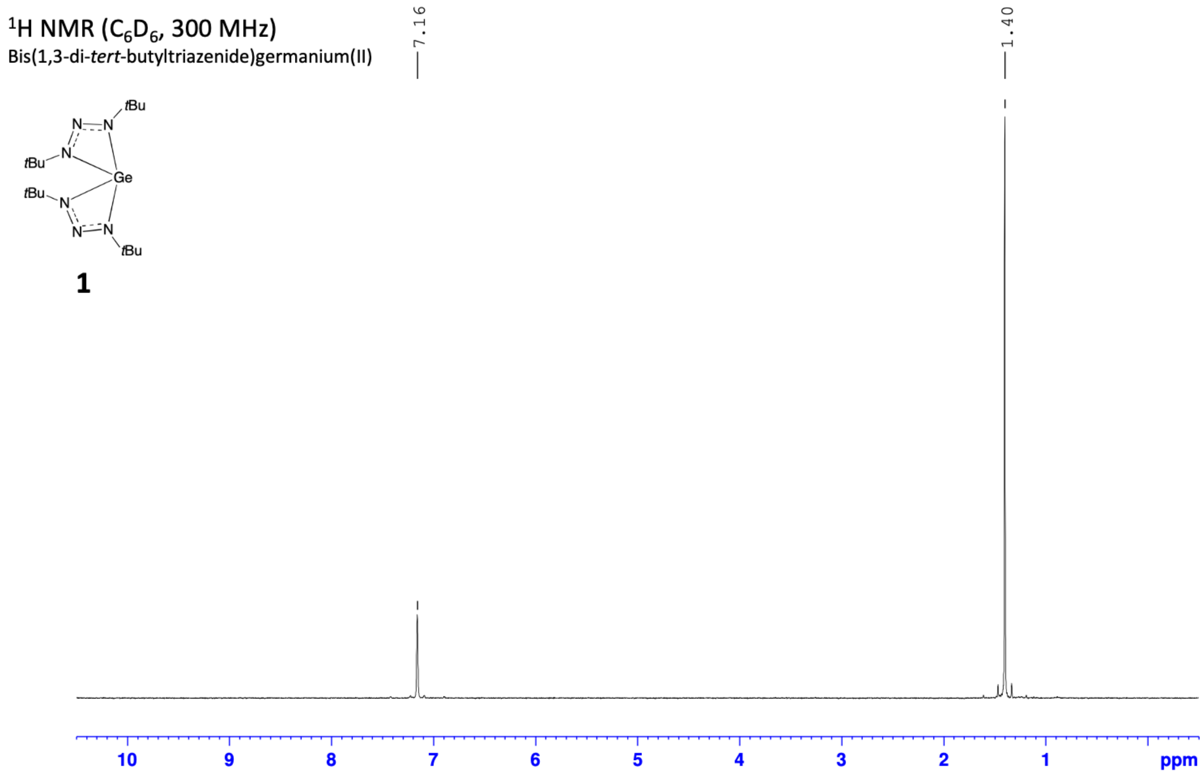


Figure S3: $^{1}H$ NMR (300 MHz) spectrum of **1** in $C_6D_6$.

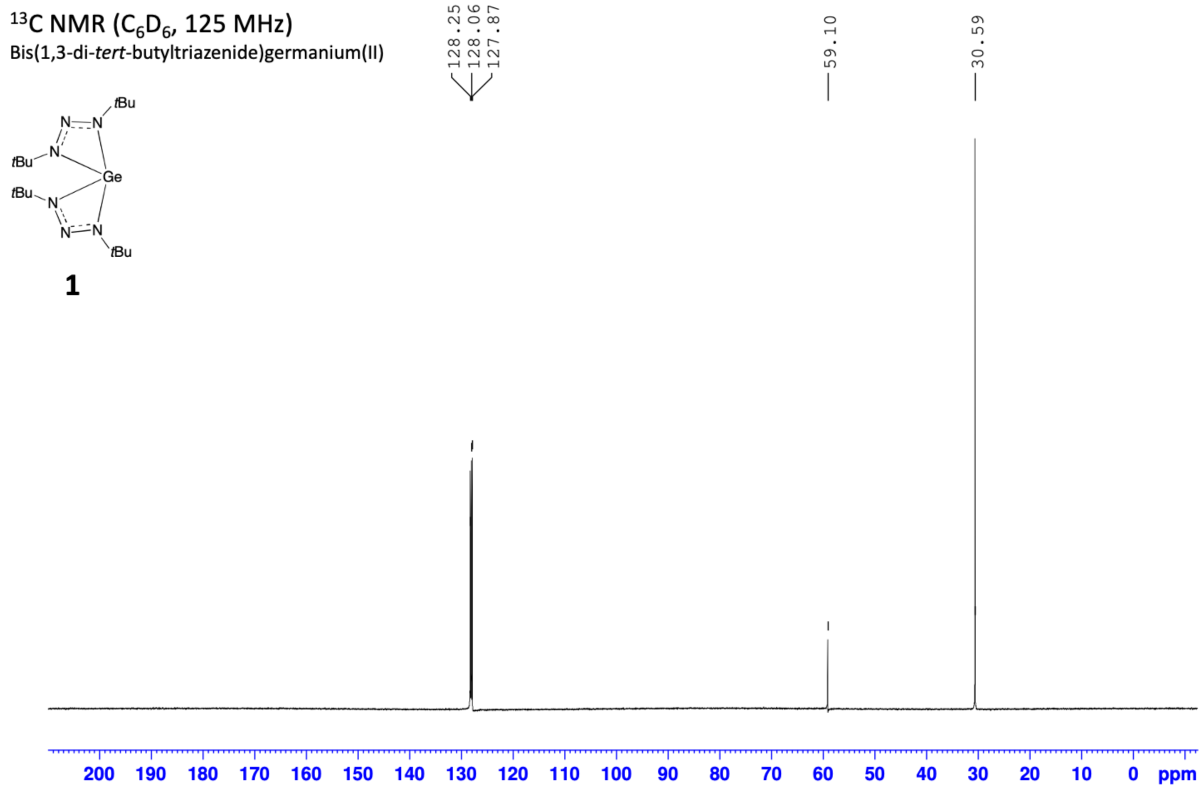


Figure S4: $^{13}C$ NMR (125 MHz) spectrum of **1** in $C_6D_6$.

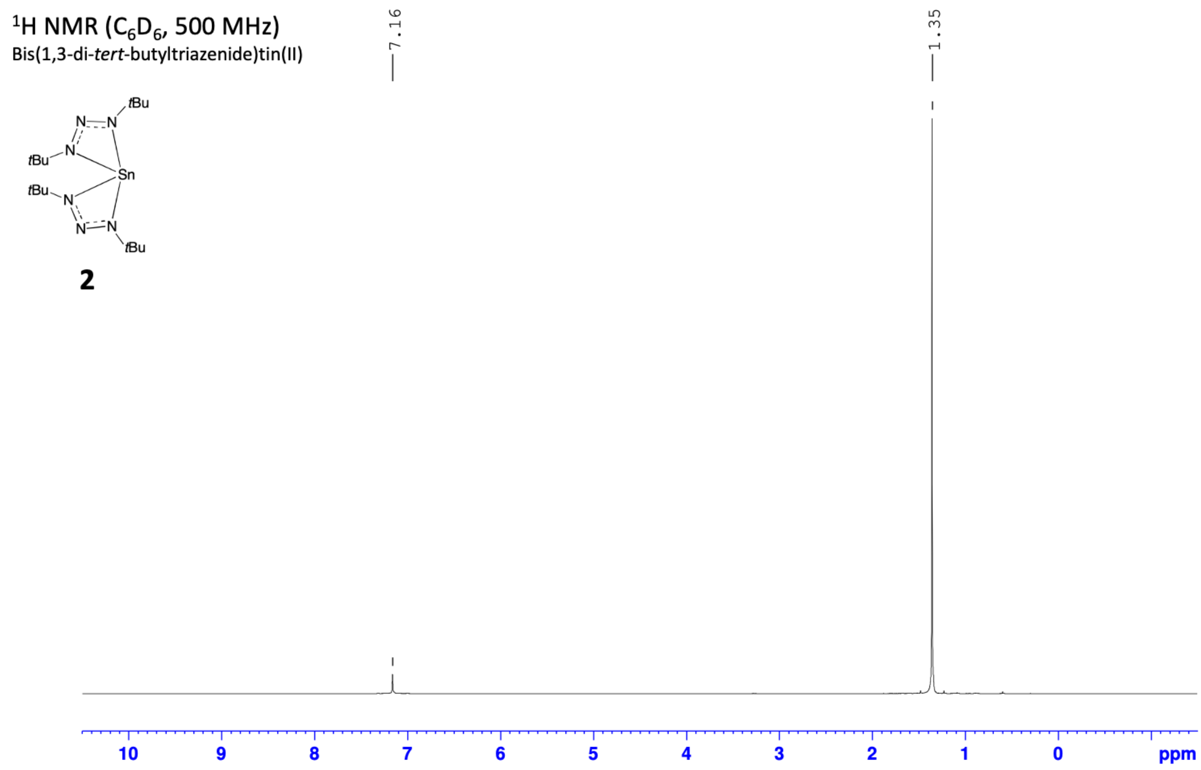


Figure S5: $^1$H NMR (500 MHz) spectrum of **2** in $C_6D_6$.

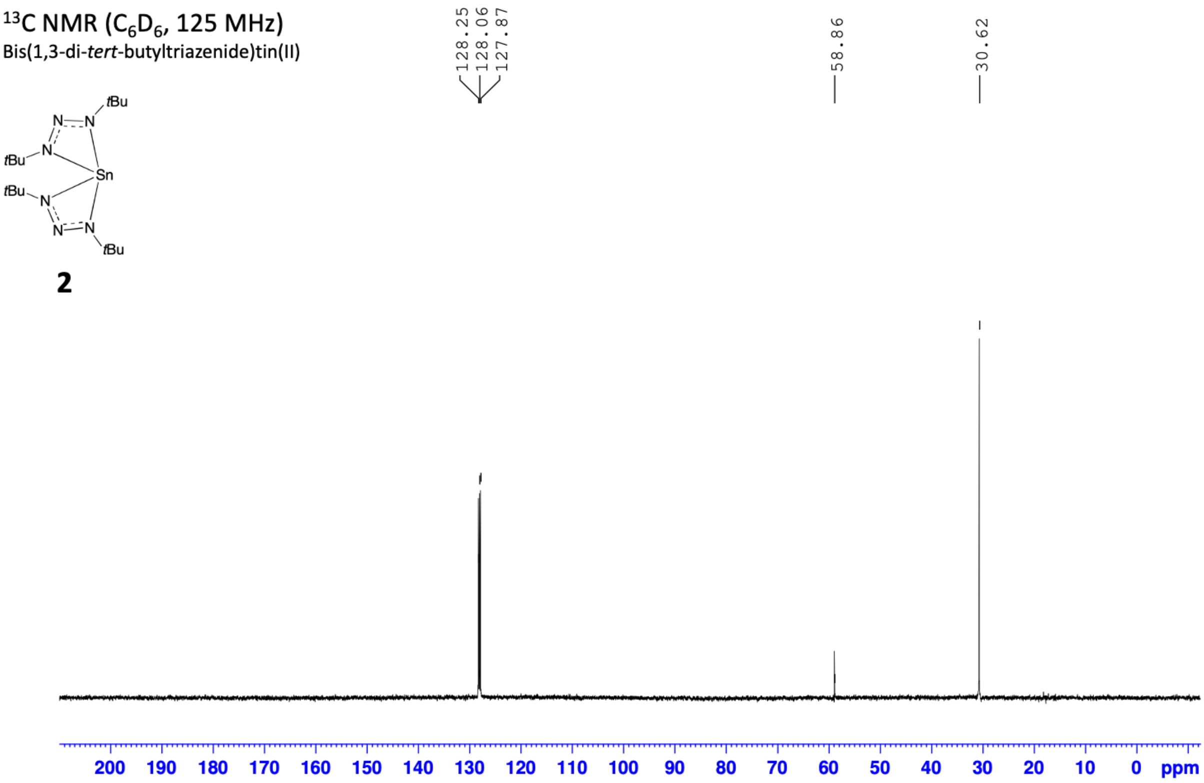


Figure S6: $^{13}$C NMR (125 MHz) spectrum of **2** in $C_6D_6$.

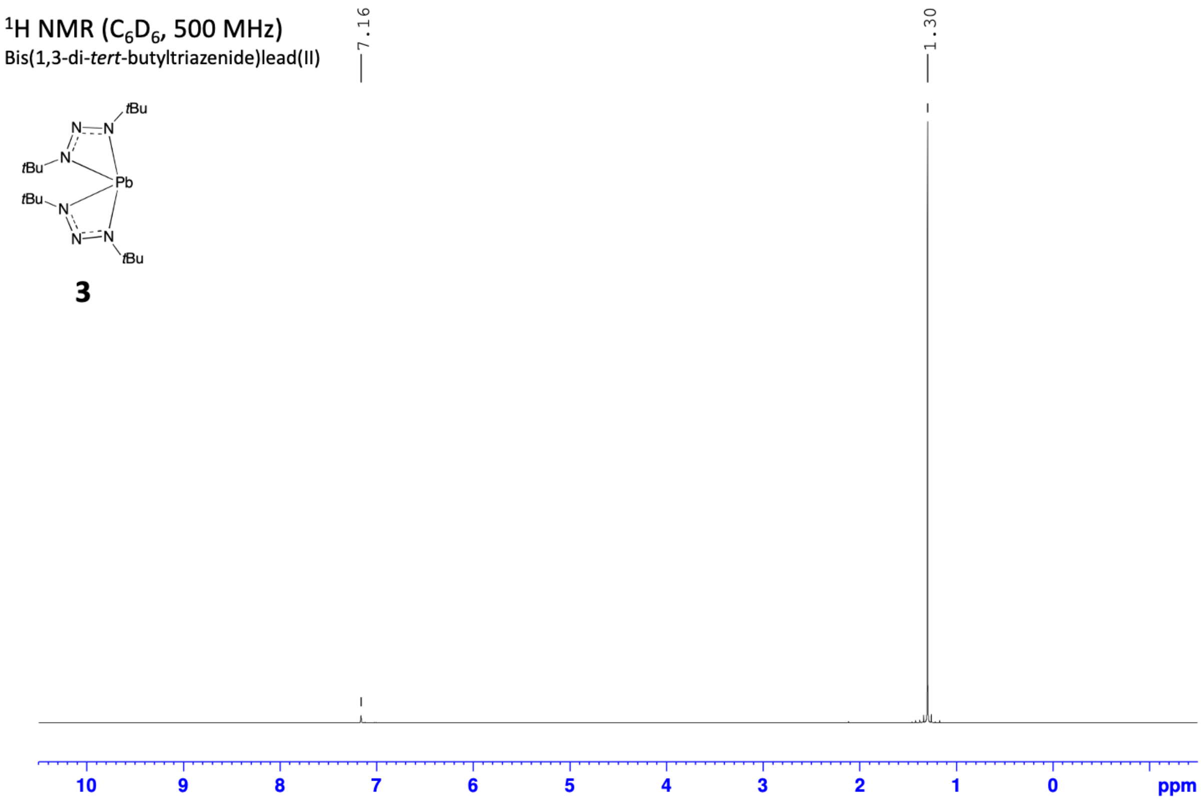


Figure S7: $^{1}$H NMR (500 MHz) spectrum of **3** in $C_6D_6$.

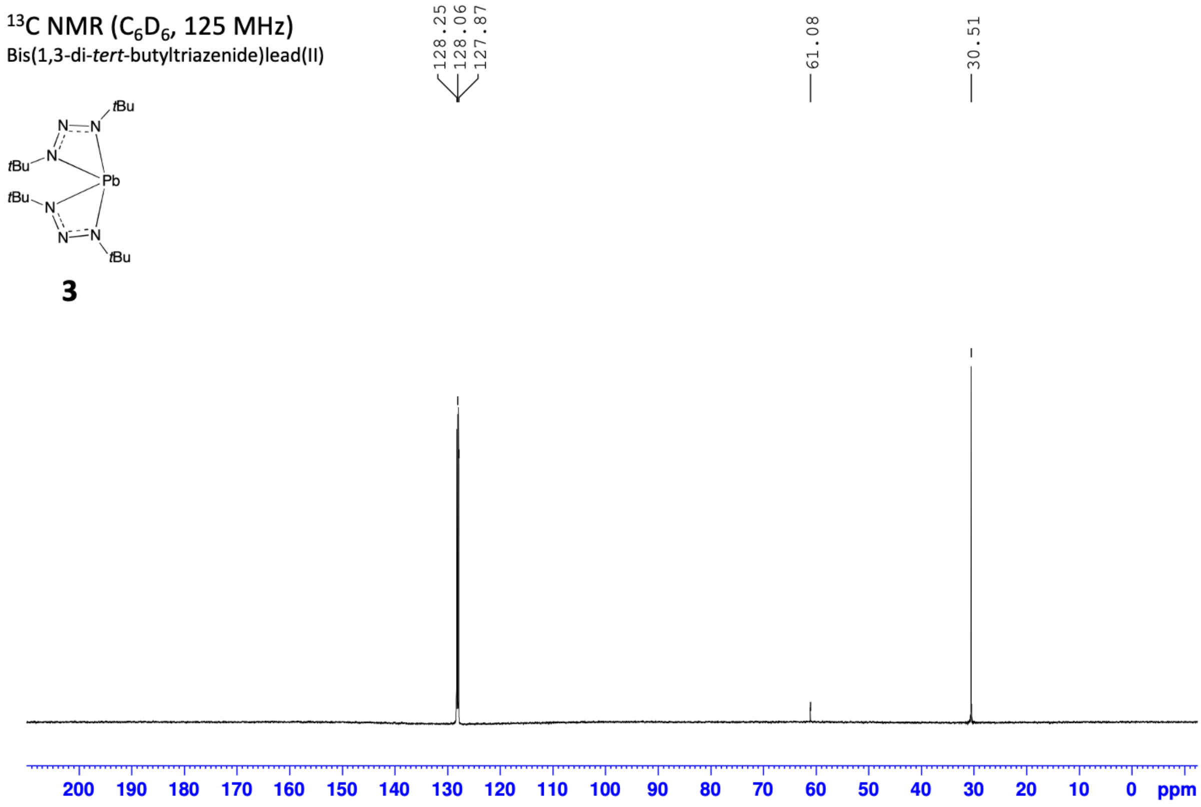


Figure S8: $^{13}$C NMR (125 MHz) spectrum of **3** in $C_6D_6$.

# Thermogravimetric Analysis

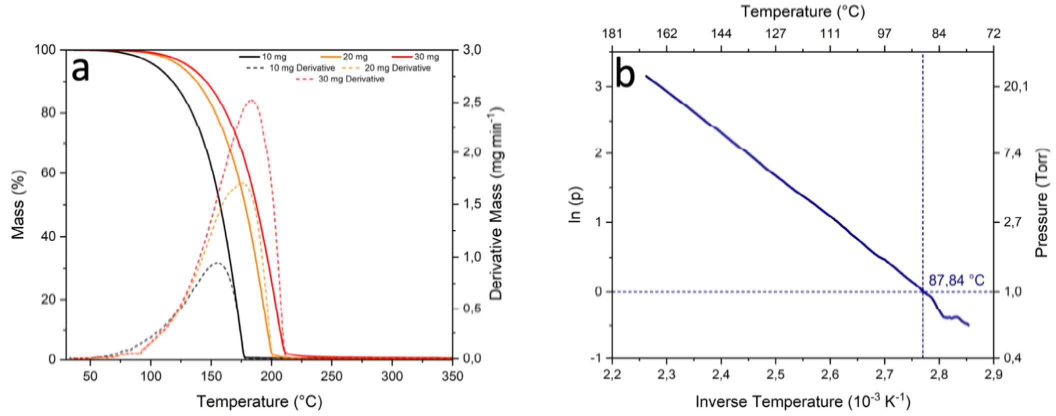


Figure S9: TGA of **1** (a) and Clausius-Clapeyron plot of **1** for derived from TGA data using 10 mg sample size (b).

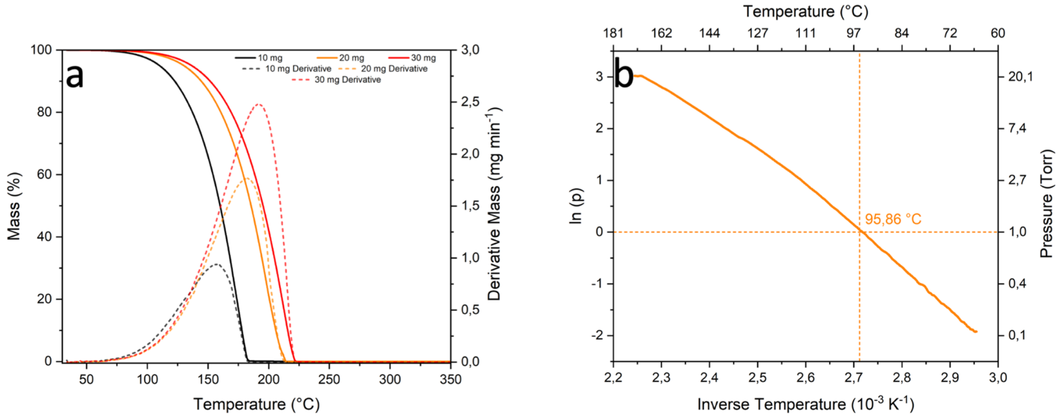


Figure S10: TGA of **2** (a) and Clausius-Clapeyron plot of **1** for derived from TGA data using 10 mg sample size (b).

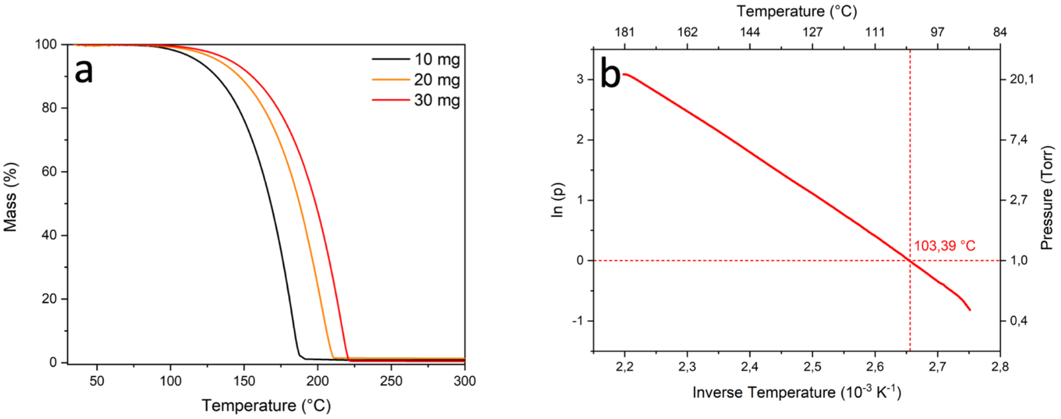


Figure S11: TGA of **3** (a) and Clausius-Clapeyron plot of **1** for derived from TGA data using 10 mg sample size (b).

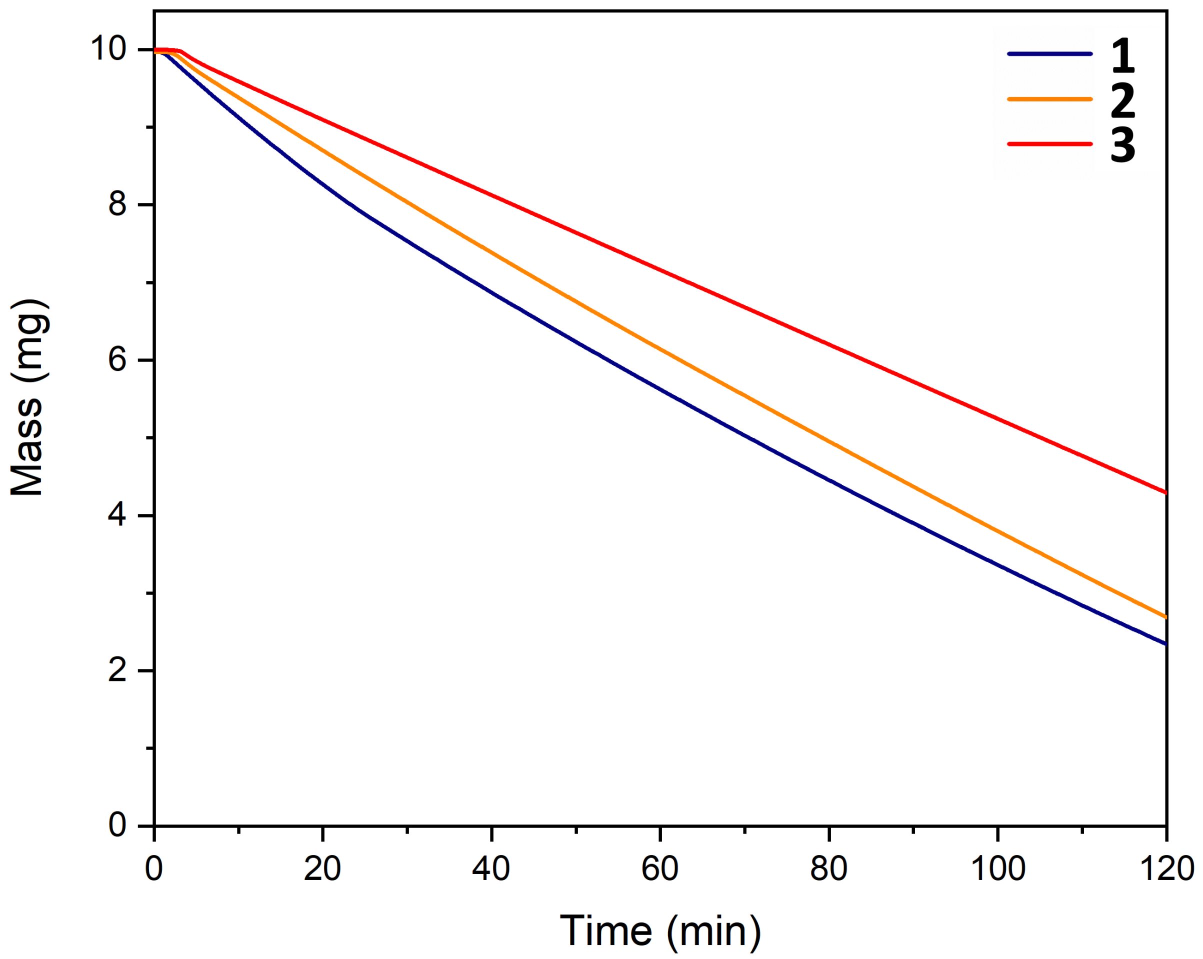


Figure S12: Isothermal TGA of **1**–**3** at 90 °C.

## Differential Scanning Calorimetry

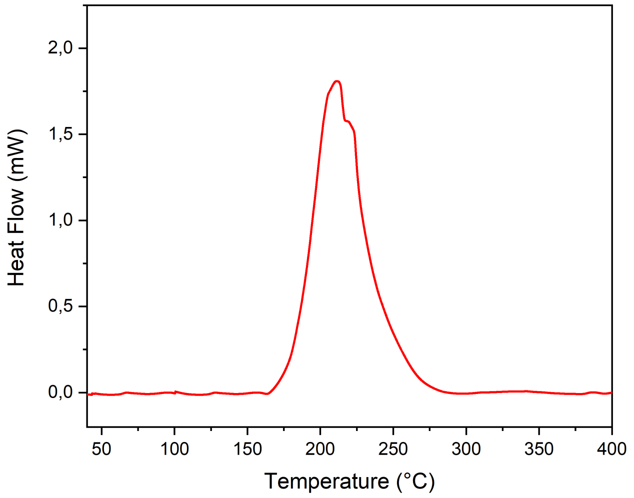


Figure S13: Base line corrected DSC of **1**.

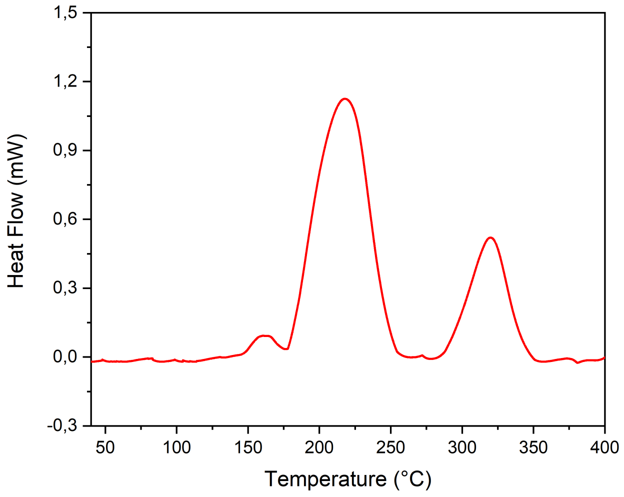


Figure S14: Base line corrected DSC of **2**.

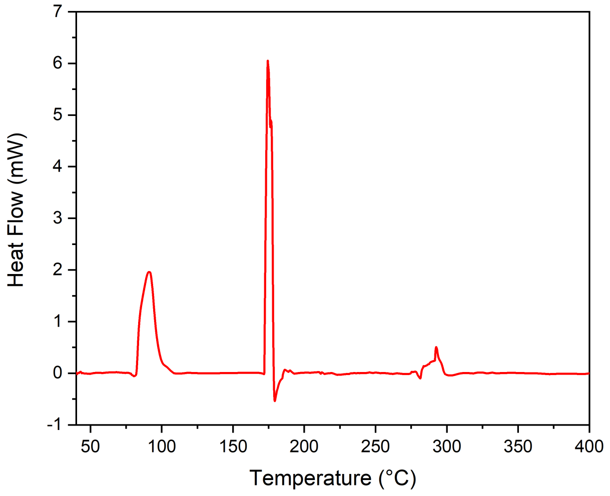


Figure S15: Base line corrected DSC of **3**.

# EI-MS Data

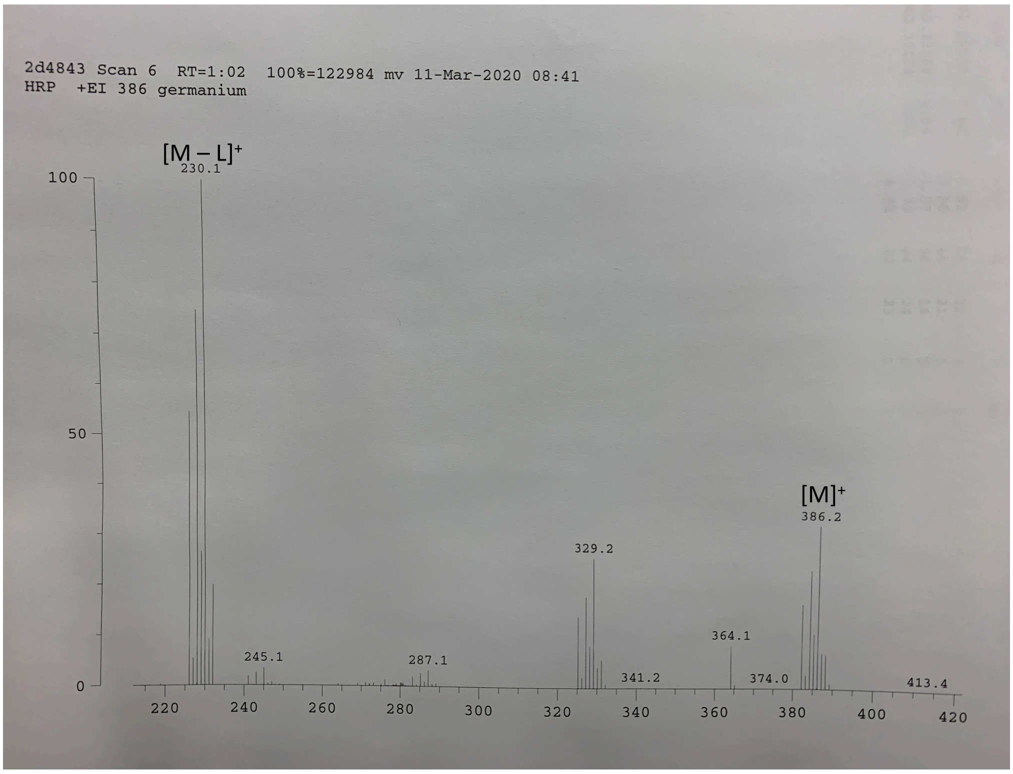


Figure S16: EI-MS of **1**.

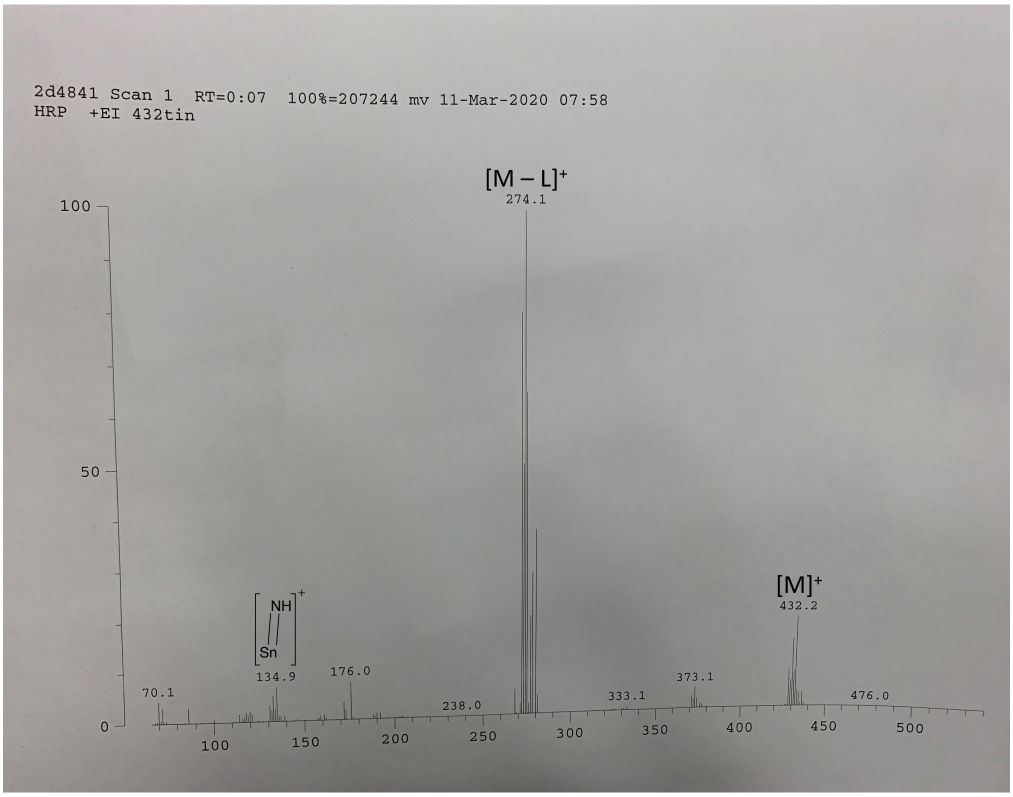


Figure S17: EI-MS of **2**.

## X-ray Crystallographic Analysis

Table S1: Bond lengths (Å) and bond angles (°) for **1** from structure determination by X-ray crystallography and quantum chemical DFT calculations.

| | XRD | DFT |
|---|---|---|
| Ge1–N1 | 1.995 | 2.036 |
| Ge1–N2 | 2.217 | 2.276 |
| N1–N3 | 1.310 | 1.297 |
| N2–N3 | 1.301 | 1.280 |
| N1–C1 | 1.477 | 1.478 |
| N2–C2 | 1.479 | 1.482 |
| N1–Ge1–N1 | 98.15 | 96.48 |
| N1–Ge1–N2 inter | 98.25 | 94.77 |
| N1–Ge1–N2 intra | 59.64 | 58.24 |
| N2–Ge1–N2 | 147.92 | 141.12 |
| N1–N3–N2 | 107.35 | 109.54 |

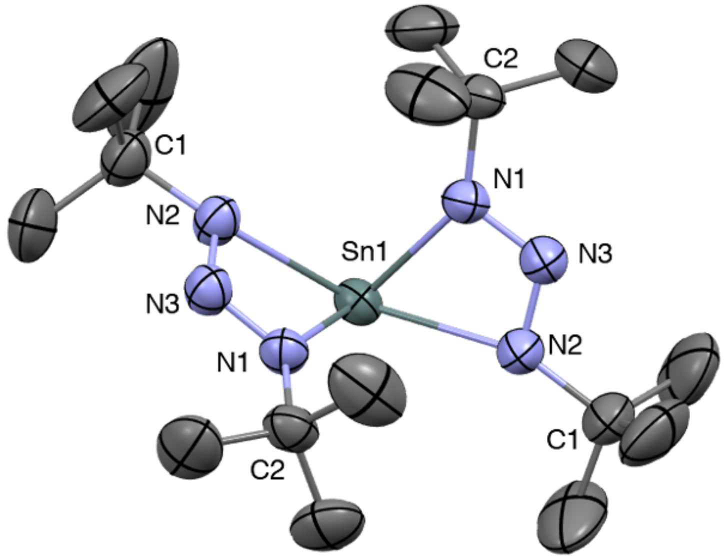


Figure S18: Crystal structure of **2**, thermal ellipsoids are depicted at 50% probability level and all hydrogens were removed for clarity.

Table S2: Bond lengths (Å) and bond angles (°) for **2** from structure determination by X-ray crystallography and quantum chemical DFT calculations.

| | XRD | DFT |
|---|---|---|
| Sn1–N2 | 2.207 | 2.248 |

| | | |
|---|---|---|
| Sn1–N3 | 2.388 | 2.423 |
| N1–N2 | 1.295 | 1.291 |
| N1–N3 | 1.293 | 1.280 |
| N2–C2 | 1.486 | 1.475 |
| N3–C1 | 1.471 | 1.479 |
| N2–Sn1–N2 | 93.99 | 92.18 |
| N3–Sn1–N3 | 133.39 | 131.31 |
| N2–Sn1–N3_inter | 92.05 | 90.91 |
| N2–Sn1–N3_intra | 54.87 | 54.02 |
| N2–N1–N3 | 110.27 | 111.59 |

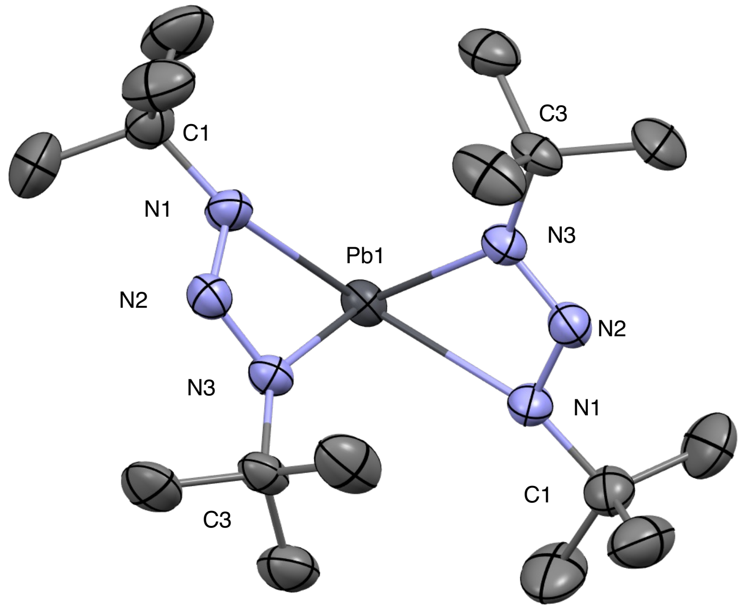


Figure S19: Crystal structure of **3**, thermal ellipsoids are depicted at 50% probability level and all hydrogens were removed for clarity.

Table S3: Bond lengths (Å) and bond angles (°) for **3** from structure determination by X-ray crystallography and quantum chemical DFT calculations.

| | XRD | DFT |
|---|---|---|
| Pb1–N1 | 2.503 | 2.423 |
| Pb1–N3 | 2.328 | 2.248 |
| N1–N2 | 1.292 | 1.291 |
| N2–N3 | 1.291 | 1.280 |
| N1–C1 | 1.476 | 1.475 |

| | | |
|---|---|---|
| N3–C3 | 1.472 | 1.479 |
| N1–Pb1–N1 | 129.61 | 131.31 |
| N3–Pb1–N3 | 92.13 | 92.18 |
| N1–Pb1–N3_inter | 91.01 | 90.91 |
| N1–Pb1–N3_intra | 52.54 | 54.02 |
| N1–N2–N3 | 112.21 | 111.59 |

Geometry index, $\tau_4'$, is defined as:

$$\tau_4' = \frac{\beta - \alpha}{360° - 109.5°} + \frac{180° - \beta}{180° - 109.5°},$$

where α and β are the two widest N–M–N angles and β > α. For compound **1**, the widest angles are 98.25 and 147.92° (Table S1). Substituting α and β for 98.25 and 147.92°, respectively in the equation for calculating $\tau_4'$ gives:

$$\tau_4' = \frac{147.92 - 98.25}{360° - 109.5°} + \frac{180° - 147.92}{180° - 109.5°} \approx 0.65.$$

Similar calculations for compound **2** using α = 93.99° and = β 133.39° (Table S2) yields $\tau_4'$ = 0.82, and for compound **3** using α = 92.13° and = β 129.61° (Table S3) yields $\tau_4'$ = 0.86.

## Computational Calculations

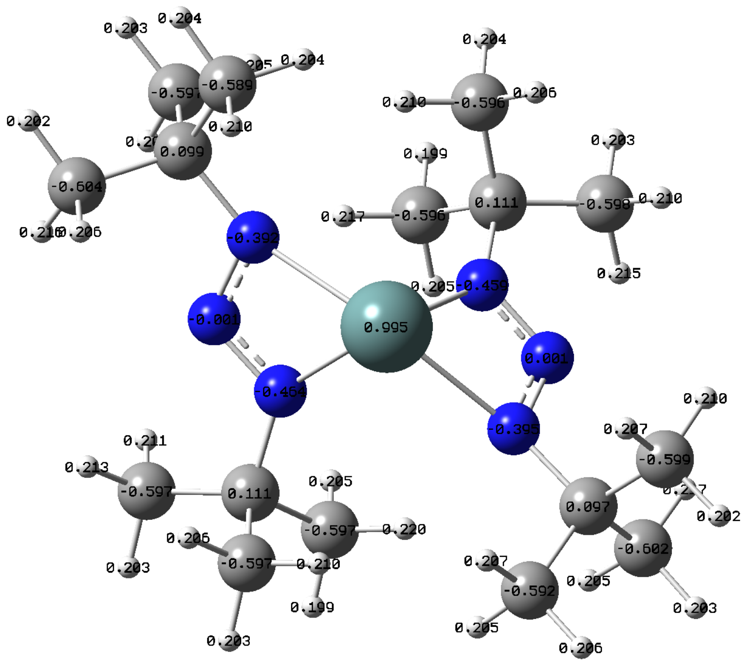


Figure S20: NBO charges for **1**.

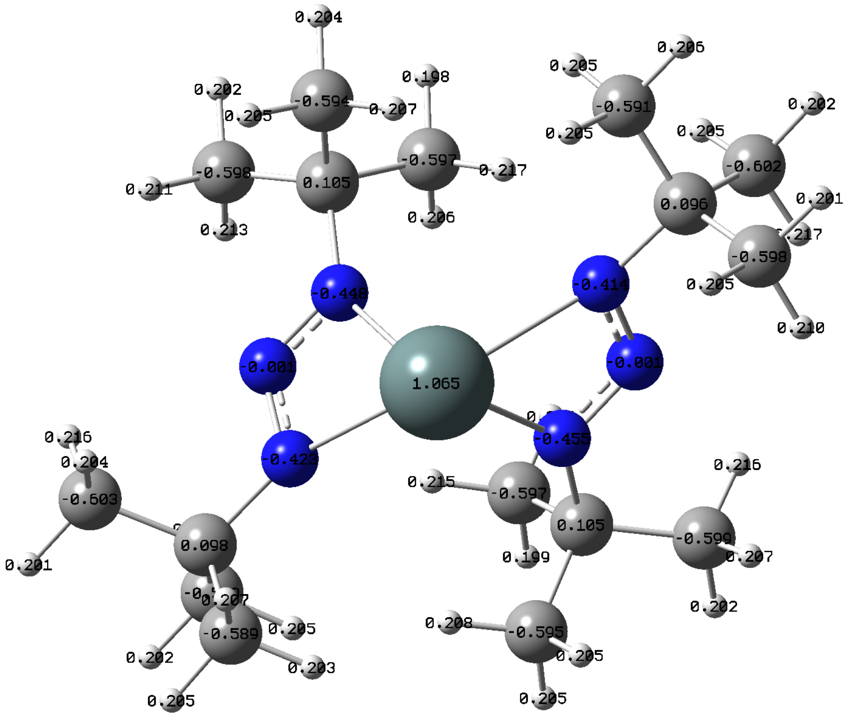


Figure S21: NBO charges for **2**.

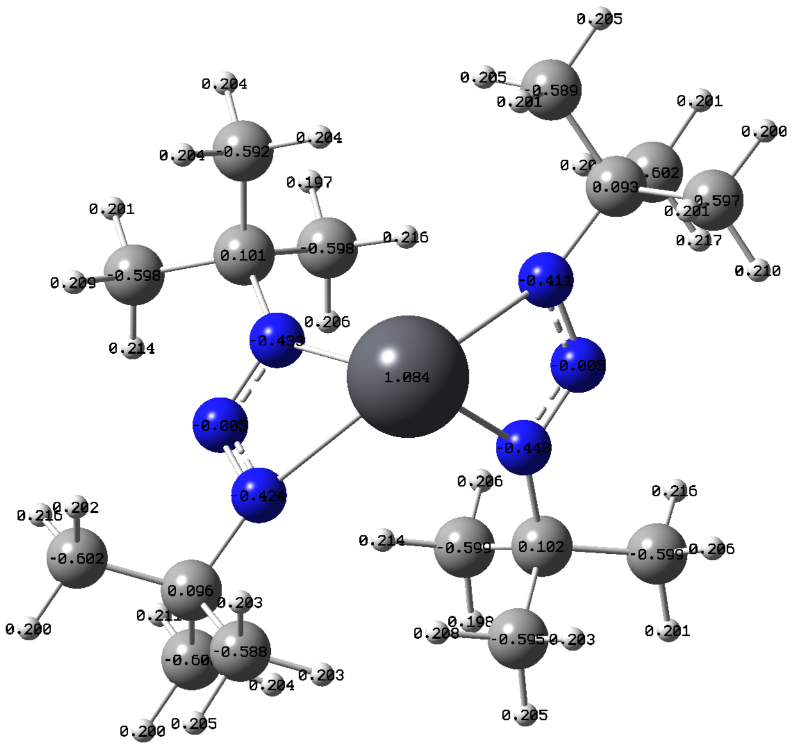


Figure S22: NBO charges for **3**.
